# Supplementary material for: Improved WOA and its application in feature selection
Source: PLoS One. 2022 May 19;17(5):e0267041. doi: 10.1371/journal.pone.0267041 (PMC9119564; doi:10.1371/journal.pone.0267041)
Supplement: Attachment — Submitted filename: Response to Reviewers.pdf [file pone.0267041.s002.pdf]

Response to Reviewers

Reviewer #1: This paper presents a method called Improved WOA and Its Application in Feature Selection.

1. The parameters used in all algorithms are given in Table 3. It should be stated why these parameter levels are used/chosen. The performances are highly dependent on the chosen levels and for obtaining best solutions one should determine the optimal parameter set for each algorithm.

Answering the question: There are two reasons why this paper chooses to use the algorithm parameters given in Table 3：

①The parameter set selected in this paper is the best parameter selection given in the original paper.

②Best choice after many experiments.

References are as follows:

[1] Faris, H., Hassonah, M. A., Ala'M, A. Z., Mirjalili, S., & Aljarah, I. (2018). A multi-verse optimizer approach for feature selection and optimizing SVM parameters based on a robust system architecture. Neural Computing and Applications, 30(8), 2355-2369.

[2] Agrawal, P., Abutarboush, H. F., Ganesh, T., & Mohamed, A. W. (2021). Metaheuristic Algorithms on Feature Selection: A Survey of One Decade of Research (2009-2019). IEEE Access, 9, 26766-26791.

[3] Asuncion, A., & Newman, D. (2007). UCI machine learning repository. http://archive.ics.uci.edu/ml.

[4] Zhao, W., Wang, L., & Zhang, Z. (2019). Atom search optimization and its application to solve a hydrogeologic parameter estimation problem. Knowledge-Based Systems, 163, 283-304.

[5] Heidari, A. A., Mirjalili, S., Faris, H., Aljarah, I., Mafarja, M., & Chen, H. (2019). Harris hawks optimization: Algorithm and applications. Future generation computer systems, 97, 849-872.

[6] Mirjalili, S. (2015). Moth-flame optimization algorithm: A novel nature-inspired heuristic paradigm. Knowledge-based systems, 89, 228-249.

[7] Mirjalili, S., Mirjalili, S. M., & Hatamlou, A. (2016). Multi-verse optimizer: a nature-inspired algorithm for global optimization. Neural Computing and Applications, 27(2), 495-513.

[8] Mirjalili, S., Gandomi, A. H., Mirjalili, S. Z., Saremi, S., Faris, H., & Mirjalili, S. M. (2017). Salp Swarm Algorithm: A bio-inspired optimizer for engineering design problems. Advances in Engineering Software, 114, 163-191.

2. Would you mind please share the codes to check the performance and data.

Answering the question: I'm very sorry to tell you that because the code used in this article needs to be used again in the team's follow-up research, the code cannot be shared, but the experimental data obtained through the code can be fully disclosed. (All data used in this paper has been uploaded to the review website).

3. The English writing should be further polished for readability.

Answering the question: In order to improve the readability of English writing, we consulted the corresponding writing experts, and combined with the experts to carefully read and grammatically correct the paper. For the revised paper, please refer to the revised manuscript.

4. The introduction paragraph should be presented more extensively.

Answering the question: In order to explain the feasibility of this study more broadly, two aspects have been added.

①Discussed and added new meta-heuristic optimization algorithm.

② The research and application of other intelligent optimization algorithms in feature selection are discussed and added

For details, please refer to the Introduction section of the revised manuscript.

5.The paragraph “Conclusions” should be enlarged highlighting the innovative contribution of the paper. Please clarify research in conclusions in modest way. Conclusions can be expanded to get clear understating of major findings.

Answering the question: In response to this problem, we have added the conclusion part of the paper. There are two additions:

①The innovation and advantages and disadvantages of the method proposed in this paper.

②Future research work progress. It consists of three parts, They are the theoretical analysis system and evaluation system of the meta-heuristic algorithm, and the community communication module; how to reduce the time complexity of IWOAIKFS and how to build a large data set preprocessing system based on IWOAIKFS.

For details, please refer to the Conclusions section of the revised manuscript.

6. it will be good to provide pros and cons of new proposed method.

Answering the question: In this paper, we propose three improved algorithms: IWOA, IKNN and IWOAIKFS, in response to your opinion, this article gives the advantages and disadvantages of the three improved algorithms, as follows:

Advantage of IWOA: Enhances the exploration ability and fast convergence ability of the algorithm in the solution space, and has better convergence performance and searching performance on most unimodal and multimodal functions

Disadvantage of IWOA: It is not suitable for all function optimization. For multimodal functions, the convergence speed is slow and the time complexity is slightly higher.

Advantage of IKNN: It can better distinguish the similarity between samples, and has better classification performance and robust performance.

Disadvantage of IKNN: High time complexity.

Advantage of IWOAIKFS: A better best feature subset can be selected.

Disadvantage of IWOAIKFS: High time complexity.

The last, I am very grateful to your comments for the manuscript. Your valuable suggestions make my manuscript better. Best wishes to you.

Reviewer #2: The manuscript, in its present form, contains several weaknesses. Adequate revisions to the following points should be undertaken to justify the recommendation for publication.

1: The previous work section is petite. I recommend adding new meta-heuristic algorithms to this section as well (Remora optimization algorithm, African vulture optimization algorithm, gorilla troops optimization algorithm, etc.).

Answering the question: In response to this problem, this article adds two parts to the introduction:

①Added new meta-heuristic optimization algorithm. For example: Remora optimization algorithm, African vulture optimization algorithm, Gorillas troop optimization algorithm, Wild horse optimizer, Binary chimp optimization algorithm, Arithmetic optimization algorithm, Aquila optimizer, etc.

② The research and application of other intelligent optimization algorithms in feature selection are added.

For details, please refer to the Introduction section of the revised manuscript.

2: Please add the system specifications used for the evaluation as well as the programming language.

Answering the question:

System: 64bit Windows 10

CPU: Intel(R) Core (TM) i7-5557U

Main frequency: 3.10GHz; RAM: 8G

Platform: Matlab2020b and Python 3.9

3: Use Friedman statistical test to better evaluation of a proposed method.

Answering the question: In response to this problem, in order to better evaluate the effectiveness of the method proposed in this paper, this paper adds Wilcoxon test and Friedman test to the IWOA algorithm, see Section 5.2.3 of the revised manuscript for details. Added Friedman test to the IWOAFS algorithm, see Section 5.4.4 of the revised manuscript for details. The test results are as follows, ***for a detailed explanation, see Sections 5.2.3 and 5.4.4 of the revised manuscript.***

①Wilcoxon test of IWOA

Table 5. *p*-value of the Wilcoxon test for the optimization results of IWOA and other algorithms based 8 benchmark functions (*p*>=0.5 are in bold).

F	IWOA vs. ASO	IWOA vs. GWO	IWOA vs. HHO	IWOA vs. MFO	IWOA vs. MVO	IWOA vs. SSA	IWOA vs. TSA	IWOA vs. WOA
				D=30				
F1	2.06E-184	5.21E-41	1.31E-25	3.94E-317	1.81E-310	2.60E-281	1.04E-84	1.15E-33
F2	4.85E-233	3.66E-40	2.64E-35	9.72E-320	8.75E-315	4.15E-308	3.64E-86	1.75E-30
F3	2.78E-64	8.08E-05	9.71E-19	1.86E-119	1.57E-72	7.67E-62	1.19E-10	3.47E-171
F4	8.62E-05	4.04E-24	2.07E-48	**NA**	**NA**	**NA**	9.82E-305	2.50E-149
F5	3.71E-305	1.64E-89	4.16E-03	1.99E-321	**NA**	1.23E-312	1.00E-323	2.07E-01
F6	3.70E-199	5.01E-68	1.65E-13	1.15E-320	5.31E-314	7.93E-310	1.27E-303	9.71E-84
F7	4.80E-293	1.98E-04	1.63E-17	**NA**	**NA**	**NA**	1.59E-185	7.24E-04
F8	5.59E-153	5.08E-173	5.85E-312	9.30E-287	2.37E-290	1.11E-291	4.03E-287	9.36E-119
w|t|l	8|0|0	8|0|0	8|0|0	6|2|0	5|3|0	6|2|0	8|0|0	8|0|0
				D=100				
F1	4.06E-274	3.15E-79	3.66E-22	1.20E-322	2.25E-315	2.65E-297	1.31E-129	6.81E-30
F2	1.35E-306	1.74E-80	1.06E-34	6.29E-321	5.90E-323	1.28E-314	9.62E-134	7.85E-31
F3	3.43E-58	1.53E-28	4.24E-27	3.57E-80	3.74E-67	1.18E-57	8.70E-43	4.88E-223
F4	**NA**	1.36E-02	1.57E-43	**NA**	**NA**	**NA**	1.18E-310	**0.16992**
F5	5.63E-322	5.30E-148	9.53E-02	**NA**	**NA**	**NA**	**NA**	**0.07075**
F6	4.18E-304	9.30E-87	6.37E-15	**NA**	6.30E-320	1.64E-316	4.27E-143	2.64E-83
F7	**NA**	4.01E-95	6.86E-17	**NA**	**NA**	**NA**	9.48E-226	4.75E-02
F8	8.75E-283	2.56E-268	1.22E-313	1.78E-321	9.90E-307	9.53E-296	1.44E-289	2.96E-31
w|t|l	6|2|0	8|0|0	8|0|0	4|4|0	5|3|0	5|3|0	7|1|0	6|0|2

②**Friedman test of IWOA**

Table 6. Friedman test results of benchmark functions with different dimensions

Dim	***p*-value**	**IWOA**	ASO	GWO	HHO	MFO	MVO	SSA	TSA	WOA
30D	**6.28E-08**	**1.38**	5.38	3.88	1.88	8.25	6.88	7.38	6.38	3.63
100D	**3.03E-08**	**1.25**	5.88	4.00	2.00	8.25	7.63	7.00	5.50	3.50

③Friedman test of IWOAIKFS

Table 12. Friedman test results for datasets

***p*-Value**	**IWOAFS**	ASO	GWO	HHO	SCA	SSA	WOA
**3.66E-12**	**1.53**	2.87	3.47	2.73	4.80	6.00	6.60

4: What method have you used to transfer solutions from continuous search space to binary space? Describe in full.

Answering the question: In order to make the proposed algorithm suitable for the feature selection problem, this paper maps the continuous search space to the binary space. The main method is to take 1 when the algorithm fitness is greater than 0.5, and take 0 when it is less than or equal to 0.

$$x_{binary} = \begin{cases} 0, & fitness \le 0.5 \\ 1, & fitness > 0.5 \end{cases}$$

5: Please add a new section then explain the computational complexity of a proposed method.

Answering the question: In this paper, we propose three improved algorithms, namely IWOA, IKNN and IWOAIKFS, For your comments, this paper gives the time complexity of the three improved algorithms, as follows:

(1) Time complexity analysis of IWOA (Section 2.4 of the revised manuscript)

The initialization of population process needs $\mathcal{O}(N \times d)$ time, where N is the population size and d defines the dimension of a given test problem.

Calculate the a and p needs $\mathcal{O}(t_{\max})$, where $t_{\max}$ is the maximum number of iterations.

Calculate the fitness of each search agent needs $\mathcal{O}(t_{\max} \times N \times d + t_{\max})$ time.

Hence, the total time complexity of IWOA algorithm is $\mathcal{O}(t_{\max} \times N \times d + t_{\max})$.

(2) Time complexity analysis of IKNN (Section 3.3 of the revised manuscript)

The traditional KNN algorithm does not require training and can be directly used for testing. So, its time complexity is $\mathcal{O}(k \times l \times m \times n)$, Among them, *k* is the number of nearest neighbors, *n* is the number of samples, *m* is the sample feature dimension, and *l* is the number of samples to be tested.

The improved KNN algorithm needs to be trained in the original data set to find the best metric matrix **M**, and then tested. The training time complexity is $\mathcal{O}(k \times T \times m^2 \times n^2)$, and the testing time complexity is $\mathcal{O}(k \times l \times m^2 \times n)$, where *k* is the number of nearest neighbors, *T* is the number of training times, *n* is the number of samples, *m* is the sample feature dimension, and *l* is the number of samples to be tested.

(3) Time complexity analysis of IWOAIKFS (Section 4.3 of the revised manuscript)

Since IWOAIKFS is a FS method obtained by IWOA optimizing IKNN, its time complexity can be divided into 3 parts, namely IWOA time complexity, IKNN complexity and IWOA optimizing IKNN time complexity. So, the time complexity of IWOAIKFS can be obtained as $\mathcal{O}\left(k \times t_{\max} \times \left(N \times m^2 \times n \times l + m^2 \times n^2\right) + t_{\max}\right)$, where *k* is the number of nearest neighbors, *N* is the population size, $t_{\max}$ is the number of iterations, *n* is the number of samples, *m* is the sample feature dimension, and *l* is the number of samples to be tested.

6: What is the value of K in the KNN algorithm? Please explain the main reason for the K-value used.

Answering the question: In this paper, the value of K in the KNN algorithm is 5. There are two main reasons for the K value to be 5:

(1) In most of the existing literature on intelligent optimization algorithms for feature selection, K=5.

Some references are as follows:

[1] Emary, E., Zawbaa, H. M., & Hassanien, A. E. (2016). Binary ant lion approaches for feature selection. Neurocomputing, 213, 54-65.

[2] Emary, E., Zawbaa, H. M., & Hassanien, A. E. (2016). Binary grey wolf optimization approaches for feature

selection. Neurocomputing, 172, 371-381.

[3] Mafarja, M. M., & Mirjalili, S. (2017). Hybrid whale optimization algorithm with simulated annealing for feature selection. Neurocomputing, 260, 302-312.

[4] Hussien, A. G., Houssein, E. H., & Hassanien, A. E. (2017, December). A binary whale optimization algorithm with hyperbolic tangent fitness function for feature selection. In 2017 Eighth international conference on intelligent computing and information systems (ICICIS) (pp. 166-172). IEEE.

[5] Mafarja, M., Aljarah, I., Heidari, A. A., Hammouri, A. I., Faris, H., Ala'M, A. Z., & Mirjalili, S. (2018). Evolutionary population dynamics and grasshopper optimization approaches for feature selection problems. Knowledge-Based Systems, 145, 25-45.

[6] Mafarja, M., & Mirjalili, S. (2018). Whale optimization approaches for wrapper feature selection. Applied Soft Computing, 62, 441-453.

[7] Too, J., Abdullah, A. R., & Mohd Saad, N. (2019). A new quadratic binary harris hawk optimization for feature selection. Electronics, 8(10), 1130.

[8] Mafarja, M., Qasem, A., Heidari, A. A., Aljarah, I., Faris, H., & Mirjalili, S. (2020). Efficient hybrid nature-inspired binary optimizers for feature selection. Cognitive Computation, 12(1), 150-175.

[9] Neggaz, N., Houssein, E. H., & Hussain, K. (2020). An efficient henry gas solubility optimization for feature selection. Expert Systems with Applications, 152, 113364.

(2) Best choice after many experiments. For the value of K, numerical experiments are carried out with K=3, K=5, K=7 respectively. Due to the influence of the characteristics of the data set itself, it is found that the experimental results are the best when K=5.

7: Please add future work to the conclusion section and discuss it briefly.

Answering the question: Future work has been added to the conclusion section.

Although the three improved methods proposed in this paper have better performance than the original algorithm, they still have some shortcomings. For example, IWOA has poor convergence performance when dealing with high-dimensional multimodal functions, and the time complexity of IKNN and IWOAIKFS is too high. Therefore, we will conduct further research on these issues in the future, as follows.

(1) In the future, we plan to build a theoretical analysis system and evaluation system for meta-heuristic algorithms, as well as a community communication module. Due to the problem of over-using "metaphor" in the meta-heuristic algorithm, in order to better distinguish the new meta-heuristic algorithm Whether (or improving the algorithm) can promote the research in the field of optimization. the follow-up research in this paper will try to establish a theoretical analysis system and evaluation system and a community communication module for the corresponding meta-heuristic algorithm.

(2) In the future, we plan to try to reduce the time complexity of IWOAIKFS. Since IWOAIKFS is the fusion of IWOA and IKNN algorithm, and influenced by IKNN algorithm, its time complexity is much higher than that of common feature selection methods. Therefore, follow-up research will try to integrate the training and testing processes in IKNN to reduce the time complexity of the IKNN algorithm, thereby reducing the time complexity of IWOAIKFS.

(3) In the future, we plan to build a large data set preprocessing system based on IWOAIKFS. After we have built the evaluation framework of the meta-heuristic algorithm and reduced the time complexity of IWOAIKFS, we can try to build a large data set preprocessing system based on IWOAIKFS, which is used to quickly process complex data sets for faster entry into machine learning.

For details, please refer to the Conclusions section of the revised manuscript.

8: What is the main reason for choosing the Gauss/mouse Chaos map, please elaborate more

Answering the question: The more evenly distributed the initial population is in the solution space, the greater the probability that the algorithm finds the optimal value. Compared with random search strategy, chaotic search is widely used in the generation of initial population due to its randomness, ergodicity, non-repetition and other characteristics. However, different chaotic maps have different effects on the initial population of the algorithm. Therefore, in this paper, through the analysis and comparison of Rand, Gauss map, Tent map and Chebyshev map, the chaotic map suitable for the whale optimization algorithm is selected. The initial population and the original initial population generated by the three chaotic maps are shown in Fig. 1.

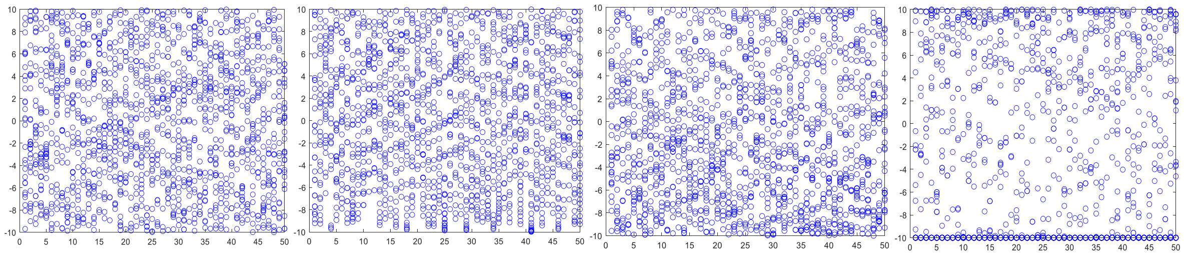

(a) Rand (b) Tent map (c) Chebyshev map (d) Gauss map

Fig.1 Initial population generated by 3 kinds of maps in 1000 iterations

In Fig.1, Figures 1(a),1(b),1(c), and 1(d) represent the original initial whale population, the initial whale population generated by Tent mapping, the initial whale population generated by Chebyshev mapping, and the initial whale population generated by Gauss Generated initial whale population. As can be seen from Figure 1, from the point of view of the generation of the initial whale population, the whale population generated by Gauss map is more evenly distributed in space, which provides a better guarantee for the global optimization of the algorithm.

For details, please refer to the Section 2.2.1 of the revised manuscript.

9: Use other criteria to evaluate the results

Answering the question: In the feature selection method, the commonly used evaluation criteria include the number of features selected, the average classification accuracy of the feature subset, the standard deviation and the convergence curve, and these evaluation criteria have been given in the manuscript, so no other criteria will be used to evaluate. But in order to better demonstrate the effectiveness of the proposed method, we draw a boxplot of the classification accuracy of all optimizers under 15 datasets for 30 independent experiments. In Fig. 12, the lower quartile (Q_1) represents lower values, the upper quartile (Q_3) represents higher values, and the red line in the box represents the median value. It can be seen from Fig. 12 that IWOAIKFS ranks first in performance among all algorithms, and has the best performance in 15 datasets.

For details, please refer to the Section 5.5.3 of the revised manuscript.

10: All the sections and subsections must be included in the text, Such as :(IWOA comparative experiment, etc.).

Answering the question: Modified as required, see revised draft for details.

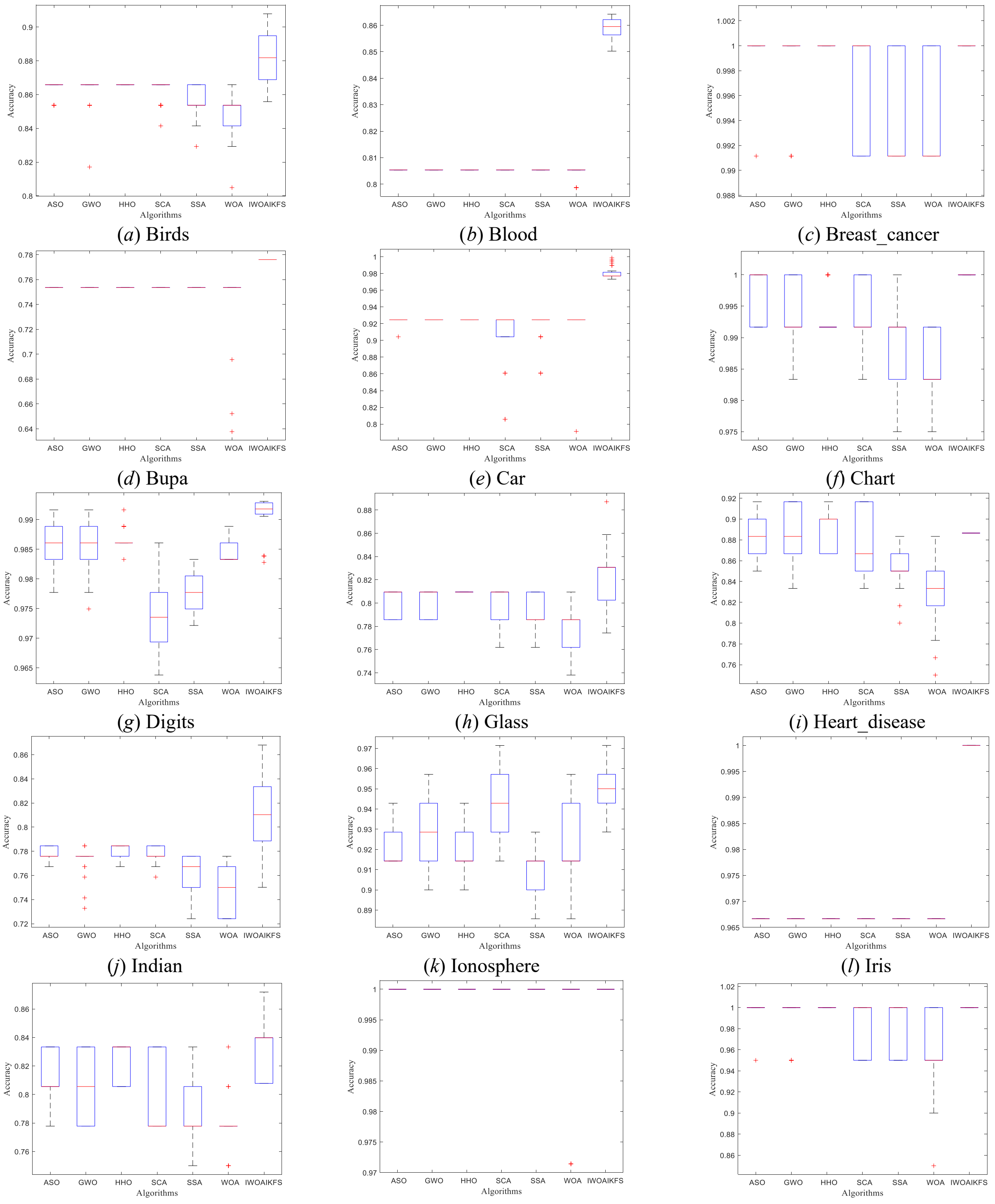

(*a*) Birds (*b*) Blood (*c*) Breast_cancer

(*d*) Bupa (*e*) Car (*f*) Chart

(*g*) Digits (*h*) Glass (*i*) Heart_disease

(*j*) Indian (*k*) Ionosphere (*l*) Iris

(*m*) Planning (*n*) Wine (*o*) Zoo

Fig. 12. Boxplot of IWOAIKFS versus other algorithms over all datasets ($k = 5$).

The last, I am very grateful to your comments for the manuscript. Thank you very much for taking time out of your busy schedule to read our manuscript and for giving us many valuable suggestions that enrich our manuscript. best wishes to you!
